# Reproduction of hemangioma by infection with subgroup J avian leukosis virus: the vertical transmission is more hazardous than the horizontal way

**DOI:** 10.1186/1743-422X-10-97

**Published:** 2013-03-27

**Authors:** Yan Lin, Jing Xia, Yang Zhao, Fuyan Wang, Songcheng Yu, Nianli Zou, Xintian Wen, Sanjie Cao, Yong Huang

**Affiliations:** 1 College of Veterinary Medicine, Sichuan Agricultural University, 46# Xinkang Road, Ya’an, Sichuan 625014, People’s Republic of China; 2 Key Laboratory of Animal Disease and Human Health of Sichuan Province, Sichuan Agricultural University, Ya’an, Sichuan, People’s Republic of China

## Abstract

**Background:**

Clinical cases of hemangioma associated with subgroup J avian leukosis virus (ALV-J) have been reported in commercial chicken layer flocks since 2006. We attempted to reproduce hemangioma through experimental infection with ALV-J to evaluate viral pathogenicity in layer birds and their progenies.

**Results:**

Body weight and indexes for immune organs of chickens infected with ALV-J strain SCDY1 were lower than those in controls. Proliferation of lymphocytes was observed in many tissues, and viral integration was detected in the genome of lymphocytes at 14 days post-infection, along with virus shedding. ALV-J was also efficiently transmitted from eggs to progenies. Embryo hatchability and progeny mortality were lower than those for controls. The efficiencies of virus shedding and virus integration in the lymphocytes of progenies were higher than those in parents.

**Conclusions:**

ALV-J is able to inhibit the growth of infected chickens, and causes damage to immune organs. Vertical transmission of ALV-J appears to be more deleterious than horizontal transmission.

## Background

Avian leukosis virus subgroup J (ALV-J) was first isolated from chickens located in Great Britain in 1988 [1]. The isolated virus was different from classical ALV subgroups A, B, C, D and E, as determined by neutralization tests, interference assays and sequence comparisons of the envelope gene [2]. ALV-J mainly induces myelocytomatosis in chickens, and diseases associated with ALV-J have caused huge economic losses worldwide since the 1990s [3]. In China, the type of neoplasm induced by ALV-J is predominantly myeloma leucosis (ML) [4]. Since 2006, clinical cases of hemangioma associated with ALV-J have been reported in commercial layer flocks [5,6], and caused significant economic losses to farmers [5,7]. Hemangiomas are vascular tumors characterized by abnormal growth of endothelial cells from capillary blood vessels [8]. They cause bleeding in chickens, and sometimes lead to death of the affected animal. Additionally, hemangiomas cause immunosuppression and lead to decreases in animal weight, egg production, fertility and hatchability [9,10].

ALV-J can be transmitted vertically and horizontally [11], with horizontal transmission more efficient than for ALVs in other subgroups [3]. To determine the pathogenicity of ALV-J associated with hemangioma, experimental infections need to be conducted. At current there are only two published reports regarding experimental infections of ALV-J [12,13], with studies related to vertical transmission of ALV-J in chickens not apparent in the literature.

In this study, experimental infection with an ALV-J strain associated with hemangioma and isolated from layer flocks in Sichuan Province was conducted. Investigation into the characteristics of ALV-J vertical transmission was also conducted.

## Results

### Physical affects of experimental infection

The body weights of chickens in the two groups were not obviously different before animals were 35 days old. Most chickens in the infected group became emaciated compared with the control chicken as time progressed. From around 70 days post-infection (d.p.i), the body weights of animals in the infected group were significantly lower than in the control group (*P* < 0.01; Table 1).

**Table 1:** Comparison of influence of SCDY1 and negative control on body weight and relative weight of immune organs

		**14d**	**35d**	**70d**	**112d**	**140d**	**168d**	**196d**
Body weight (g)	SCDY1	82.6 ± 3.80	285.10 ± 21.01	1146.13 ± 53.30^A^	1219.13 ± 53.47^A^	1282.27 ± 48.31^A^	1331.70 ± 58.30^A^	1366.27 ± 60.45^A^
	NC	89.47 ± 6.80	303.97 ± 10.81	1468.93 ± 39.1^B^	1523.57 ± 88.24^B^	1586.67 ± 84.20^B^	1628.87 ± 60.94^B^	1671.40 ± 59.46^B^
Spleen relative weight	SCDY1	1.03 ± 0.08^A^	1.52 ± 0.17	1.40 ± 0.31	1.95 ± 0.09^A^	1.95 ± 0.18 ^A^	1.82 ± 0.26 ^a^	1.68 ± 0.26 ^a^
	NC	0.49 ± 0.02^B^	1.33 ± 0.12	1.65 ± 0.06	1.63 ± 0.04^B^	1.56 ± 0.08 ^B^	1.56 ± 0.03 ^b^	1.53 ± 0.01 ^b^
Thymus relative weight	SCDY1	4.59 ± 0.53	4.47 ± 0.60 ^a^	3.12 ± 0.14 ^a^	1.66 ± 0.07^A^	1.31 ± 0.10^A^	0.82 ± 0.04^A^	0.44 ± 0.11^A^
	NC	6.46 ± 1.15	6.09 ± 0.41 ^b^	3.86 ± 0.29 ^b^	2.53 ± 0.20^B^	1.95 ± 0.21^B^	1.21 ± 0.03^B^	0.87 ± 0.08^B^
Bursa relative weight	SCDY1	2.78 ± 0.21	2.77 ± 0.10^A^	1.49 ± 0.15 ^a^	1.28 ± 0.03 ^a^	1.12 ± 0.14 ^A^	0.84 ± 0.09 ^A^	0.79 ± 0.04 ^A^
	NC	3.15 ± 0.64	3.66 ± 0.08^B^	1.18 ± 0.10 ^b^	1.00 ± 0.15 ^b^	0.65 ± 0.10 ^B^	0.34 ± 0.09 ^B^	0.25 ± 0.06 ^B^

Note: Figure in the table referred to mean ± SD; the data with different capital letters in same column show extremely significant difference; the data with different little letters in same column show significant difference; no letter in same column show no significant difference.

The relative weight of immune organs (RWIO) was calculated using the following formula:

$$\begin{array}{l} \text{RWIO}\;=\;\text{immune}\;\text{organ}\;\text{weight}\;\\ \;\times 1,000/\text{animal}\;\text{body}\;\text{weight} \end{array}$$

[14] For the spleen, the mean RWIO in ALV-J 14-day-old infected chickens (1.03) was significantly higher (P < 0.01) than that for controls (0.49). However, no significant difference (P > 0.5) in RWIOs was observed between 14 and 112 days. Once chickens were 112 days old, the RWIOs for the infected group were always higher than for the control group. For the thymus and bursa, RWIOs decreased during the course of experimental infection, with the mean RWIO for the thymus in the 35-day-old ALV-J infected group (4.47) significantly lower than that for the control group (6.09). The mean RWIO for the bursa in 35-day-old infected chickens (2.77) was lower than for the controls (3.66), but was higher than controls for chickens aged 70 days and older.

### Hemangioma in white leghorn chickens

**Figure 1:**
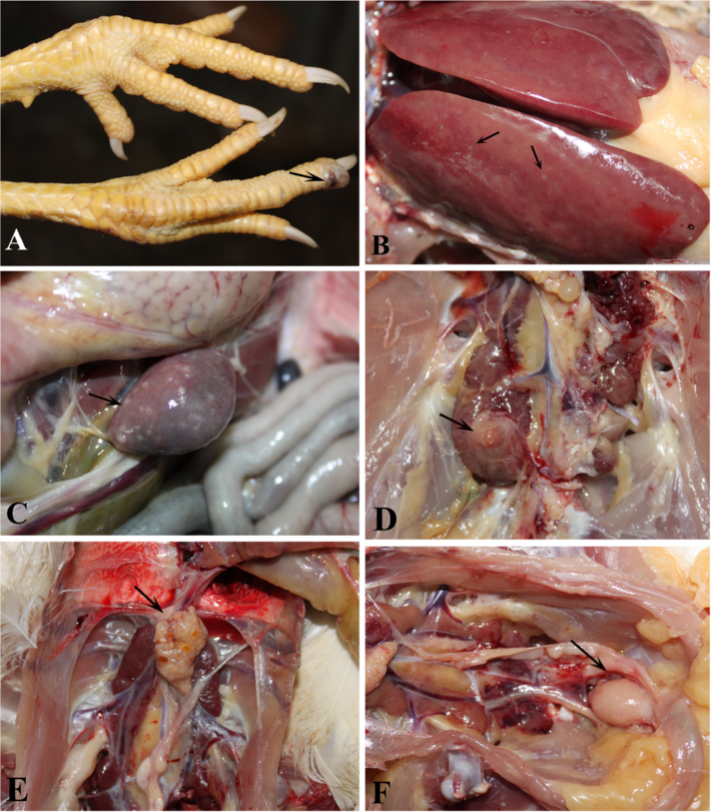
**Anatomical lesions induced by SCDY1 in SPF chickens.** (**A**) Hemangioma in the subcutaneous of the feet; (**B**) Tumour in liver; (**C**) Tumour in spleen; (**D**) Tumour in kidney; (**E**) ovaries had atrophied; (**F**) bursa had enlarged.

During the course of the experimental infection, 30 chickens were infected with no deaths reported, and 4 chickens showing signs of hemangioma. All animals were euthanized and autopsied upon completion of the experiments. The first case of skin hemangioma was observed at 140 d.p.i, and presented as a small red hemorrhagic nodule on the toes, with a diameter of about 1 cm (Figure 1A). Following autopsy, ten chickens had histopathological changes; 6 of them had small white nodules on the surface of enlarged livers, spleens and kidneys (Figure 1B, C and D). The ovaries and fallopian tubes of 4 infected chickens were poorly developed (Figure 1E), and the bursa of 5 chickens were enlarged (Figure 1F).

**Figure 2:**
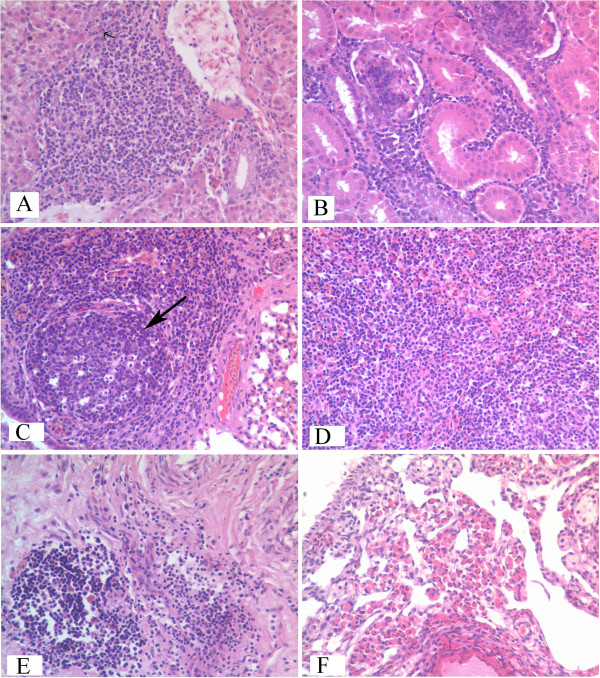
**Histopathologic observation of diseased chickens infected by ALV-J SCDY1 strain.** Diagnosis by histopathologic slices (HE 400×). All tissues were fixed in 10% formalin paraffin and embedded, and 5 μm sections were stained with haematoxylin and eosin (HE), for light microscopy, “**A**” to “**F**” refer to liver, spleen, lungs, spleens, bursas and ovary. Arrows show that multimorphology lymphocytes infiltrated patically and formed tumors (**A**, **C**), other organs were infiltrated by lymphocytes extensively and their organizations were completely destroyed (**B**, **D**, **E**), and eosinphilic granule cells infiltrated in ovary (**F**).

Histopathologically, aggressive growth of hepatic hemangiomas was observed, with a large number of lymphocytes found to have proliferated in the portal and central venous areas. The sinusoid was squeezed by the swelling of liver cells, with some liver cells degenerated (Figure 2A). Tumor cells in the kidneys, lungs, spleens and bursas originated from lymphoid cells (Figure 2B, 2C, 2D and 2E). In the atrophied ovary, a large number of eosinophilic granule cells could be seen (Figure 2F). Other tissues such as skeletal and heart muscle contained no histopathological changes. The control group did not show any histopathological changes.

### Viral DNA integration in lymphocytes and virus shedding

We found that viral DNA became integrated in the genomes of lymphocytes. Virus shedding in the cloaca was determined by enzyme-linked immunosorbent assays ELISAs). ALV-J proviral DNA in lymphocytes could be detected at 14, 112, 140, 168 and 196 d.p.i, but not at 35 and 70 d.p.i. Shed virus was detected throughout the experimental period in infected chickens. Proviral cDNA integration and virus shedding in control chickens was not observed for the entire study period (Table 2).

**Table 2:** Results of proviral cDNA detection in lymphocyte and P27 antigen detection in cloacae swabs

**Groups**	**Sample**	**14d**	**35d**	**70d**	**112d**	**140d**	**168d**	**196d**
SCDY1	Proviral cDNA	2/30^a^	0/30	0/30	2/30	6/30	6/30	12/30
	P27 antigen	6/30	10/30	8/30	6/30	6/30	8/30	12/30
Control	Proviral cDNA	0/30	0/30	0/30	0/30	0/30	0/30	0/30
	P27 antigen	0/30	0/30	0/30	0/30	0/30	0/30	0/30

^a^ Positive samples/total samples.

### Vertical transmission of ALV-J SCDY1

For the 82 embryos collected from ALV-J SCDY1-infected chickens, 4 embryos were unfertilized, 56 fertilized embryos died while hatching, and 22 chicks hatched. Of the 22 that hatched, 4 died soon after birth, 8 survived for 10 days, and 10 survived the remainder of the experimental period. Tissues from dead chickens were found to be ALV-J-positive by polymerase chain reaction (PCR; data not shown). For the 10 chicks that survived hatching and the rest of the experimental period, proviral cDNA integration in lymphocytes and virus shedding were detected from the time of hatching (1 day old; Table 3). Of the 10 animals, 6 were positive by PCR, and P27 antigen was constantly detected. Two of the chickens were sometimes positive for the presence of proviral cDNA and P27 antigen, and 2 chickens were always negative for proviral cDNA and P27 antigen. Fertilized eggs (n = 80) were collected from the control group, with 70 of these hatched chickens surviving. PCR and ELISA results for these chickens were negative for the duration of the experiment (data not shown).

**Table 3:** Results of proviral cDNA detection in lymphocyte and P27 antigen detection in cloacae swabs of the progenies

**Chicken number**	**Days**						
	**1d**	**10d**	**30d**	**60d**	**80d**	**90d**	**100d**
1	+^a^ (+)^b^	+ (+)	+ (+)	+ (+)	+ (+)	+ (+)	+ (+)
2	+ (+)	+ (+)	+ (+)	+ (+)	+ (+)	+ (+)	+ (+)
3	+ (+)	+ (+)	+ (+)	+ (+)	+ (+)	+ (+)	+ (+)
4	- (−)	- (−)	+ (+)	+ (−)	+ (−)	+ (−)	+(−)
5	- (−)	- (−)	- (−)	- (−)	- (−)	- (−)	- (−)
6	- (−)	- (−)	+ (+)	+ (+)	+ (−)	+ (−)	+ (−)
7	- (−)	- (−)	- (−)	- (−)	- (−)	- (−)	- (−)
8	+ (+)	+ (+)	+ (+)	+ (+)	+ (+)	+ (+)	+ (+)
9	+ (+)	+ (+)	+ (+)	+ (+)	+ (+)	+ (+)	+ (+)
10	+ (+)	+ (+)	+ (+)	+ (+)	+ (+)	+ (+)	+ (+)

^a^ indicate the result of proviral cDNA detection, ^b^ indicate the result of P27 antigen detection.

“+” represent positive result for proviral cDNA or P27 antigen detection, “-” represent negative result.

## Discussion

The first clinical cases of hemangioma associated with ALV-J were observed in Roman layer chickens. Typical clinical symptoms include small, well-circumscribed violaceous or red hemorrhagic nodules on the skin or abdominal organs. Our experimental reproduction of hemangioma in layer flocks demonstrated that ALV-J on its own can induce hemangioma.

In our study, we observed that ALV-J SCDY1 inhibited the growth of chickens and caused damage to immune organs. The RWIOs from the thymus of ALV-J SCDY1-infected chickens were always lower than those of controls, suggesting that ALV-J infection may inhibit the development of thymus. For the bursa, RWIOs for chickens in the infected groups were lower than for controls during the earlier periods of the study, but were elevated above control levels during the latter stages of our study. This would suggest that the development of the bursa in ALV-J-infected chickens was inhibited early, and the increase in RWIOs later might be related to the occurrence of inflammation. For the spleen, RWIOs in ALV-J-infected chickens were higher than in controls, indicating constantly active viral replication and inflammation. Histopathologically, lymphocyte proliferation was continuously occurring for the duration of the experimental period.

The genome of ALV can integrate into the chromosome of host cells during viral replication cycle, therefore PCR is used to detect proviral cDNA [15]. Samples were PCR-positive as early as 14 d.p.i in some chickens, indicating that PCR could be used for the early diagnosis of ALV-J infection. Although proviral cDNA could be detected during the early stages of infection, the occurrence of hemorrhagic nodules on the skin of infected chickens was only observed once they began to lay eggs. We speculated that the tumors induced by ALV-J might be related to hormone levels in chickens, however further studies would be needed to confirm this. In this study, 13.3% (4/30) of infected chickens had skin hemangioma, 33.3% (10/30) demonstrated histopathological changes and 40% (12/30) shed virus. These findings suggest ALV-J infection is not the only factor to induce neoplasms; with environmental conditions and individual differences also contributing to hemangioma pathogenicity. With respect to virus shedding, the P27 antigen was detected in most experimentally infected chickens at 14 d.p.i., however some of the infected chickens constantly shed virus, while others only shed virus occasionally [16]. These findings suggest to us that the mechanisms of replication employed by ALV-J *in vivo* are very complicated.

In this study, the hatchability of embryos from infected chickens and survival ratio of progeny were significantly lower than those for controls, with more than 50% of progenies from ALV-J infected chickens shedding virus upon hatching. A minority of birds, aged 1–100 days, remained negative or only occasionally positive for proviral cDNA and P27 antigen. These are strong indicators to the poultry industry that ALV-J infection can have significant deleterious effects upon layers, as the efficiency of vertical transmission is high. The elimination of ALV-J infection from breeding chickens should be considered the most urgent priority for the poultry industry worldwide.

## Conclusions

Hemangioma was solely induced by ALV-J infection, although incidence of infection was low, and integration of proviral cDNA in lymphocytes along with virus shedding could only be detected in some infected chickens. ALV-J infection inhibits the growth of chickens and damages organs of the immune system. The vertical transmission of ALV-J caused a decline in embryo hatchability and mortality of progeny due to the high efficiency of vertical transmission.

## Methods

### Virus

The ALV-J SCDY1 strain was isolated from a breeding farm in Sichuan Province (China) in 2010. The complete proviral genome has been sequenced and analyzed [17].

### Experimental infection of chickens

Embryos (n = 70) from specific pathogen-free (SPF) chickens (Merial-Vital Laboratory Animal Technology Co., Ltd, Beijing, China) were hatched, and 60 SPF chicks (1 day old) were divided randomly into two groups. Thirty were inoculated intraperitoneally with 100 μL of cell culture supernatant containing 10^6^ tissue culture infectious doses (TCID_50_) ALV-J SCDY1. The other 30 chickens were inoculated intraperitoneally with 100 μL of uninfected cell culture supernatant and served as negative controls. Chickens were kept in separate bio-safety level 2+ (BSL2+) isolators with *ad libitum* access to food and water and maintained under uniform standard management conditions. Approval for these animal studies was obtained from the Animal Care and Use Committee (ACUC) of Sichuan Agricultural University. The body weight, RWIO, integrated viral DNA in lymphocytes, virus shedding, clinical signs and gross postmortem lesions as well as mortalities were recorded for 28 weeks post-inoculation. All animals, except for 2 cocks and 6 hens in the experimental group, were euthanized at the end of the experimental period. Eggs were collected and hatched, and progenies raised for 100 days. The integration of viral DNA in lymphocytes, and level of virus shedding were recorded.

### Tissue collection and examination

Blood samples and cloaca swabs were obtained when chickens were 14, 35, 70, 112, 140, 168 and 196 days old. Three chickens were randomly selected from each group to be weighed and then autopsied after sampling. Thymus, bursa and spleen were collected to determine their weights in relation to total body weight. Liver, heart, kidney, spleen, thymus and lung with gross tumors were fixed in 10% neutral-buffered formalin, dehydrated, embedded in paraffin wax and sectioned (5 μm thickness) for histopathological examination.

### Viral DNA integration in lymphocytes

Blood samples were collected in tubes containing heparin from the wing vein. Lymphocytes were separated from whole blood using lymphocyte separation medium (Tian Jin Hao Yang Biological Manufacture Co., Ltd, Tianjin, China) according to the manufacturer’s instructions. Total DNA was extracted from lymphocytes using a sodium dodecyl sulfate (SDS) buffer supplemented with proteinase K, and phenol:chloroform:isoamylol (25:24:1). Primers designed by Smith [15] were synthesized and used in the PCR detection of ALV-J proviral DNA.

### Virus shedding

Cloaca swabs were tested for the presence of P27 antigen using an Avian Leukosis Virus Antigen Test Kit (Beijing WDWK Biotechnology Co., Ltd, Beijing, China) according to the manufacturer’s instructions. A cut-off (s/p ratio) value of 0.17 was used.

### Vertical transmission of ALV-J SCDY1

ALV-J infected chickens (196 days old) that were positive for both proviral cDNA and P27 antigen were raised separately, and 82 eggs collected and hatched. The offspring were raised to an age of 100 days in BSL2+ isolators. Blood samples and cloaca swabs were collected when birds were 1, 10, 30, 60, 80, 90 and 100 days old. Proviral cDNA and P27 antigen detection were performed as described above. Fertilized eggs (n = 80) were collected from control chickens and hatched; offspring were raised and detection of proviral DNA and P27 antigen conducted in the same way as for ALV-J experimentally infected chickens.

### Statistical analysis

Differences between the experimental and control groups were assessed by analysis of variance (ANOVA) with SAS 8.0 software, with differences considered statistically significant when *P*-values were less than 0.05.

## Competing interests

The authors declare that they have no competing interests.

## Authors’ contributions

YL participated in the design of the study, performed the experiments, interpretation of the data and wrote the manuscript. JX, YZ, FYW, SCY, NLZ, XTW, SJC, YH conceived of the study, and participated in its design and coordination and helped to draft the manuscript. All authors read and approved the final manuscript.
